# Supplementary material for: Discovery of Benzopyrone-Based Candidates as Potential Antimicrobial and Photochemotherapeutic Agents through Inhibition of DNA Gyrase Enzyme B: Design, Synthesis, In Vitro and In Silico Evaluation
Source: Pharmaceuticals (Basel). 2024 Sep 11;17(9):1197. doi: 10.3390/ph17091197 (PMC11434840; doi:10.3390/ph17091197)
Supplement: Supplementary file 1 [file pharmaceuticals-17-01197-s001.zip › pharmaceuticals-3134840-supplementary.pdf]

Discovery of Benzopyrone-Based Candidates as Potential Antimicrobial and Photochemotherapeutic Agents Through Inhibition of DNA Gyrase Enzyme B: Design, Synthesis, In Vitro and In Silico Evaluation

Akram Abd El-Haleem [1,§,*], Usama Ammar [2,§,*], Domiziana Masci [3], Sohair El-Ansary [1,4], Doaa Abdel Rahman [4,*], Fatma Abou-Elazm [5], Nehad El-Dydamony [1,§]

[1] Pharmaceutical Chemistry Department, College of Pharmaceutical Sciences and Drug Manufacturing, Misr University for Science and Technology, Al-Motamayez District, P. O. Box:77, 6th of October City, Egypt; akram.hifny@must.edu.eg (A.A.), sohir.alansary@must.edu.eg (S.E.), nehad.eldydamony@must.edu.eg (N.E.)
[2] School of Applied Sciences, Edinburgh Napier University, Sighthill Campus, 9 Sighthill Court, Edinburgh, EH11 4BN, United Kingdom; u.ammar@napier.ac.uk
[3] Department of Basic Biotechnological Sciences, Intensivological and Perioperative Clinics, Catholic University of the Sacred Heart, Largo Francesco Vito 1, 00168 Rome, Italy; domiziana.masci@unicatt.it
[4] Department of Pharmaceutical Chemistry, Faculty of Pharmacy, Cairo University, Kasr El-Aini Street, Cairo 11562, Egypt; sohir.alansary@must.edu.eg (S.E.), doaa.abdelghani@pharma.cu.edu.eg (D.A.)
[5] Department of Microbiology and Immunology, College of Pharmaceutical Sciences and Drug Manufacturing, Misr University for Science and Technology, Al-Motamayez District, P. O. Box:77, 6th of October City, Egypt; fatma.alsayed@must.edu.eg
* Correspondence: u.ammar@napier.ac.uk (U.A.); akram.hifny@must.edu.eg (A.A.); doaa.abdelghani@pharma.cu.edu.eg (D.A.)
§ These authors contributed equally to this work.

Supplementary Material

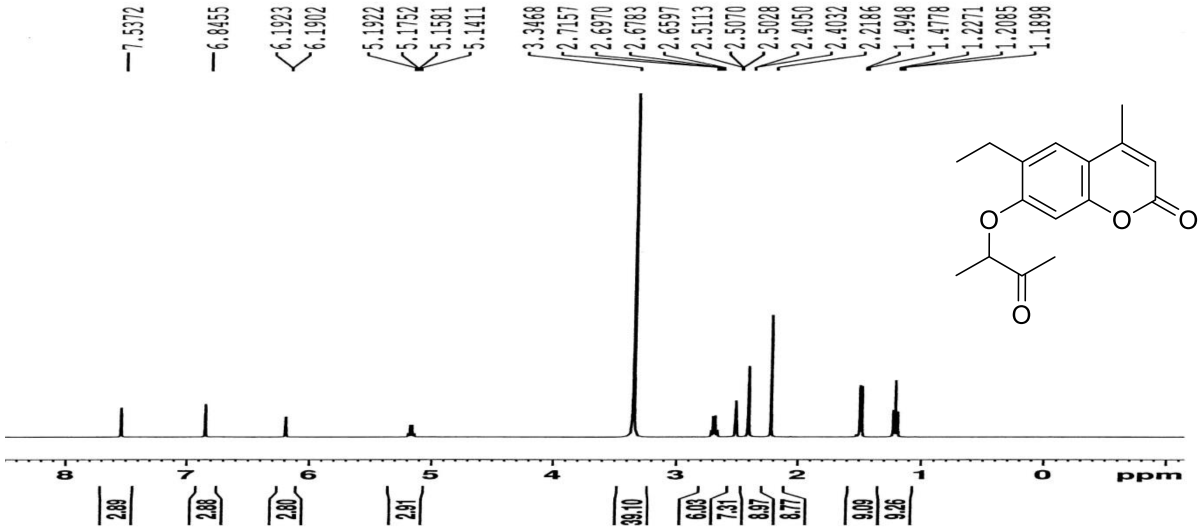

Figure S1. ^{1}H NMR spectrum (400 MHz, DMSO-d_6) of compound **2a**.

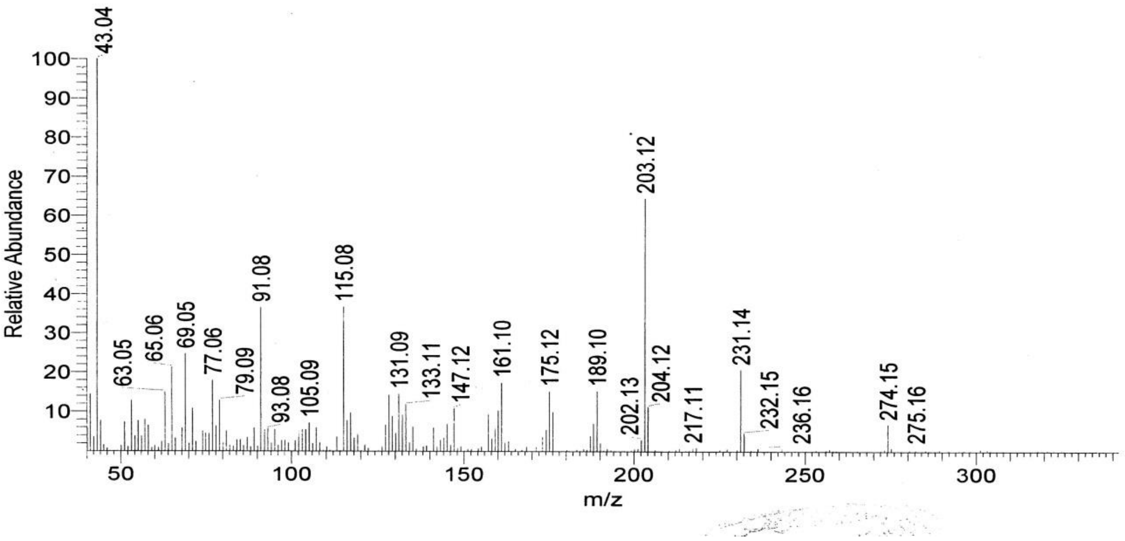

Figure S2. Mass spectrum of compound **2a.**

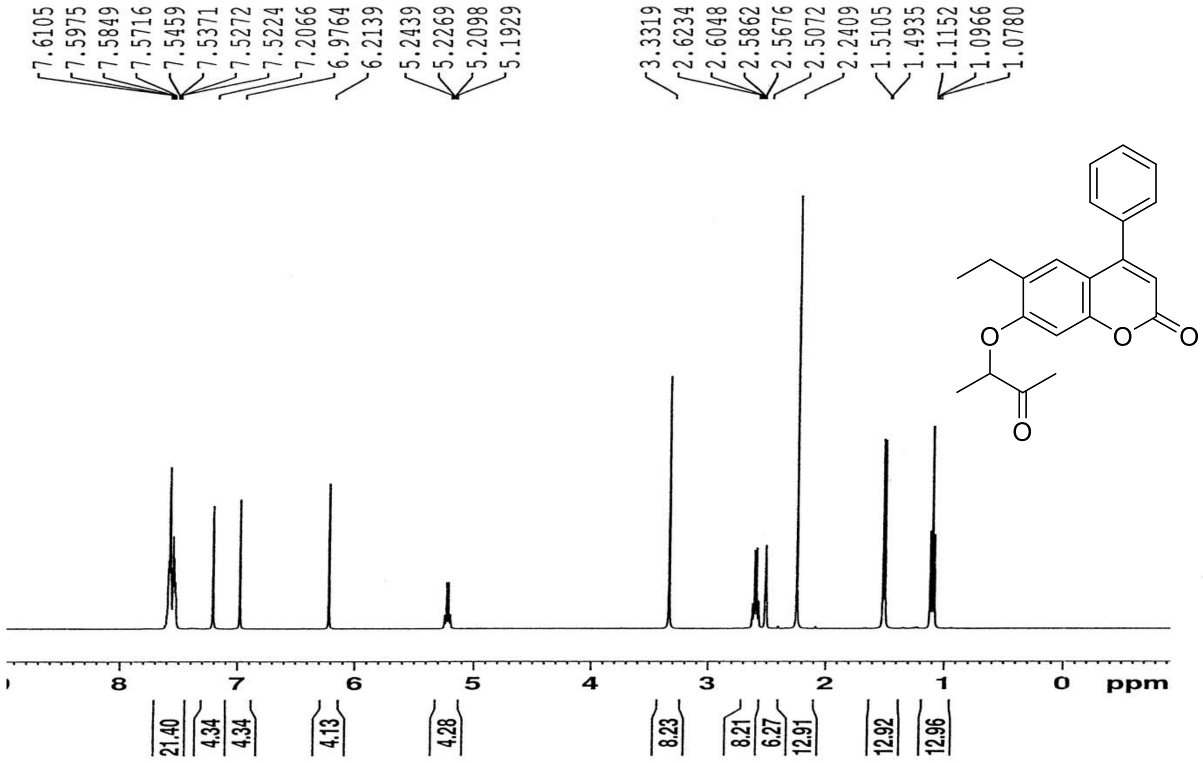

Figure S3. ^{1}H NMR spectrum (400 MHz, DMSO-d_6) of compound **2b.**

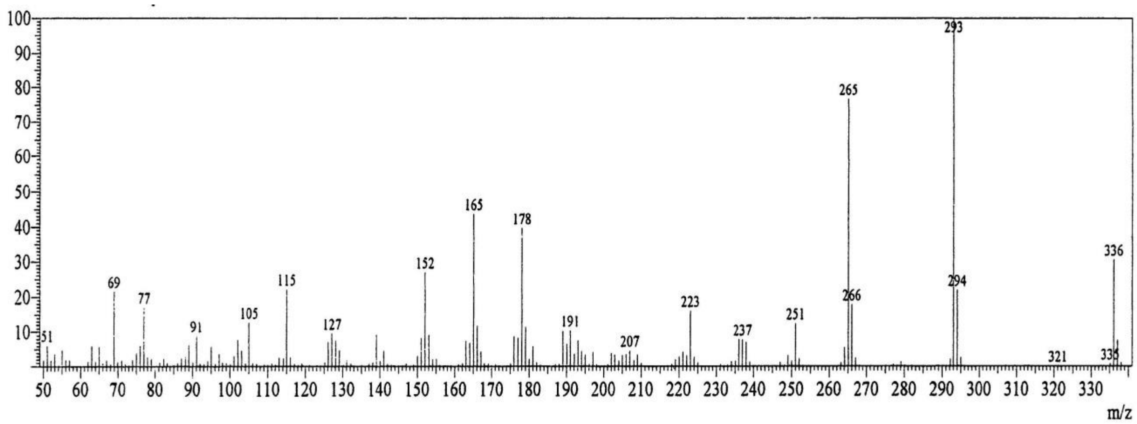

Figure S4. Mass spectrum of compound **2b**.

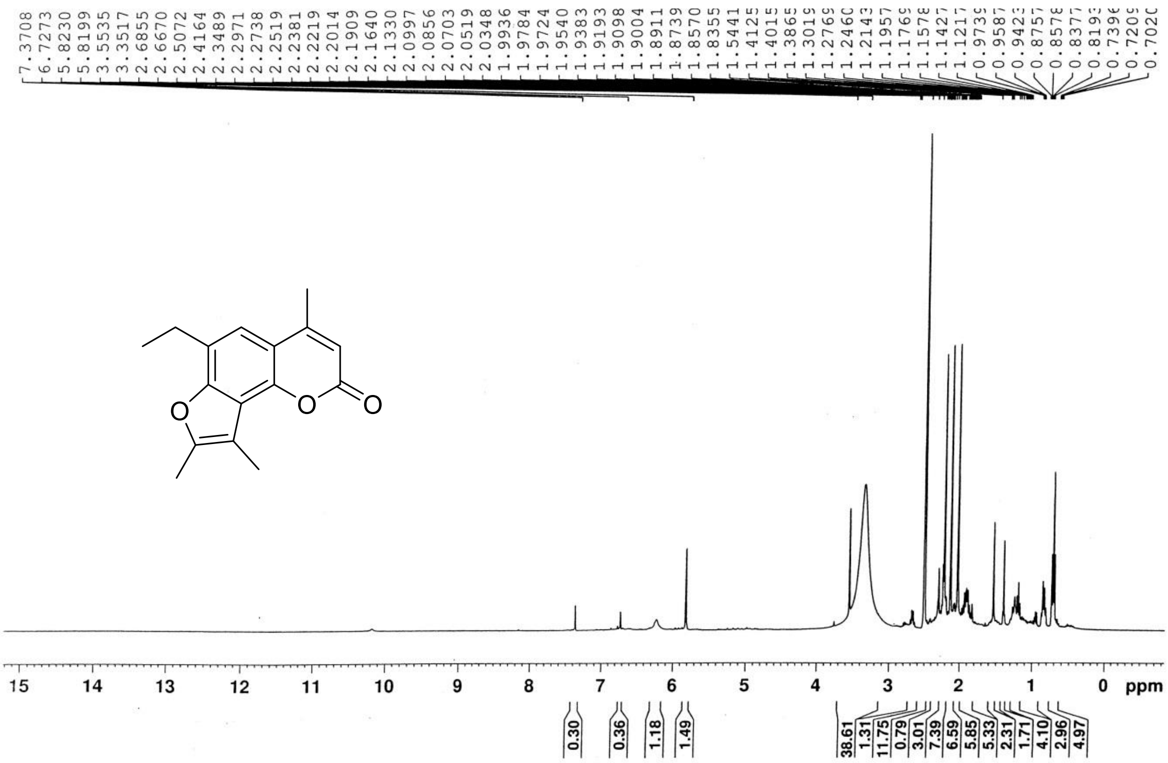

Figure S5. ^{1}H NMR spectrum (400 MHz, DMSO-d_6) of compound **3a**.

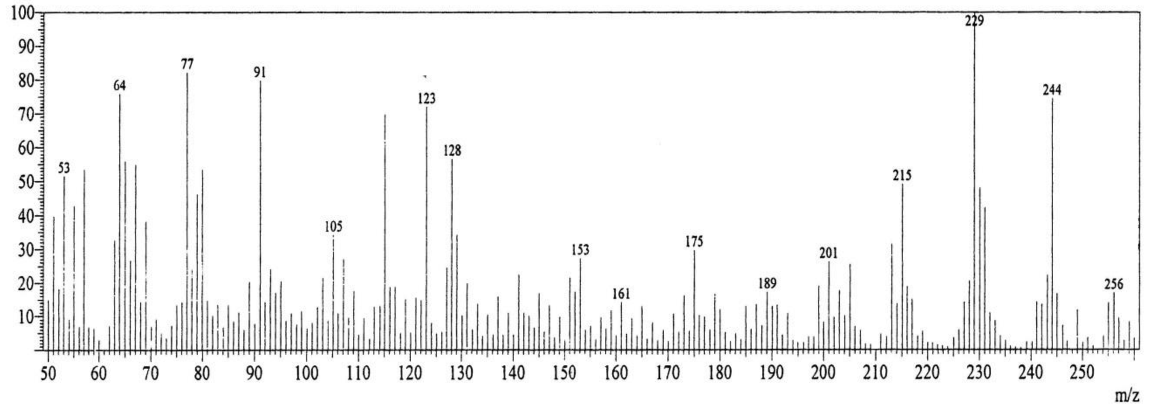

Figure S6. Mass spectrum of compound **3a**.

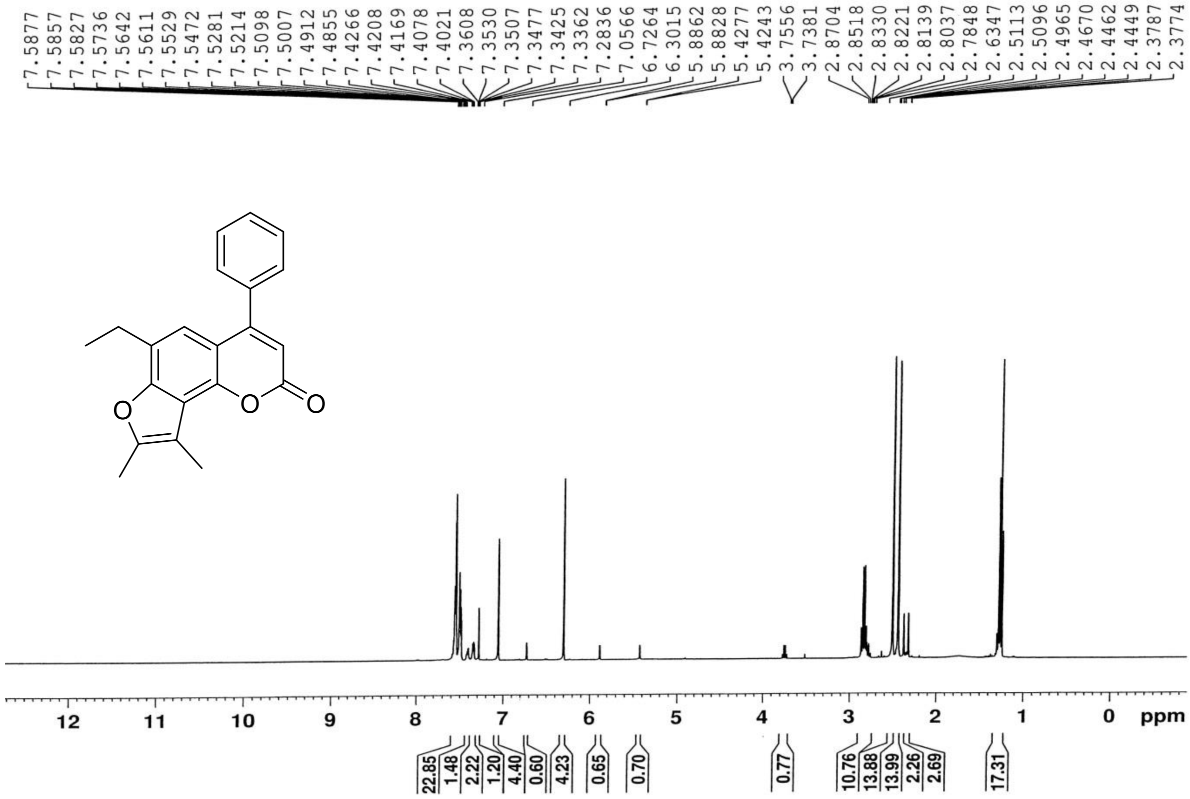

Figure S7. ^{1}H NMR spectrum (400 MHz, $CDCl_3$) of compound **3b**.

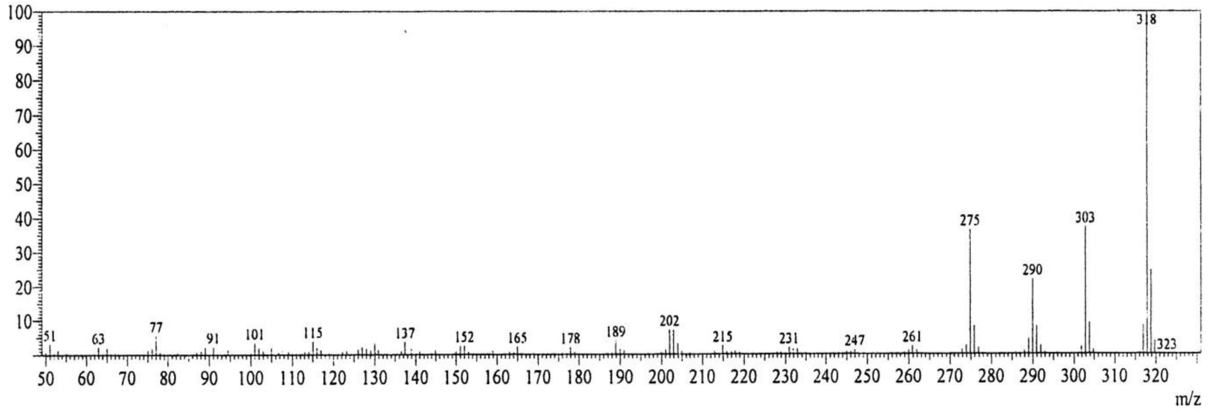

Figure S8. Mass spectrum of compound **3b**.

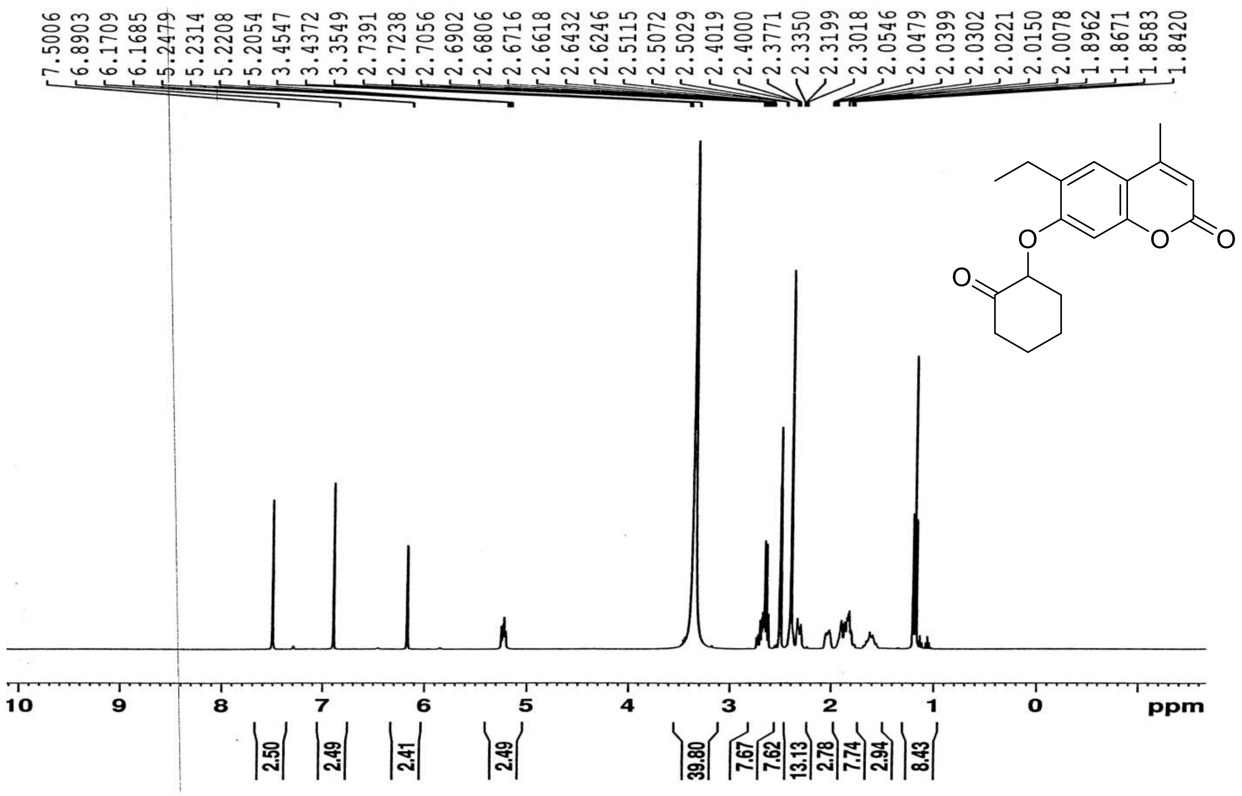

Figure S9. ^{1}H NMR spectrum (400 MHz, DMSO-d_6) of compound **4a**

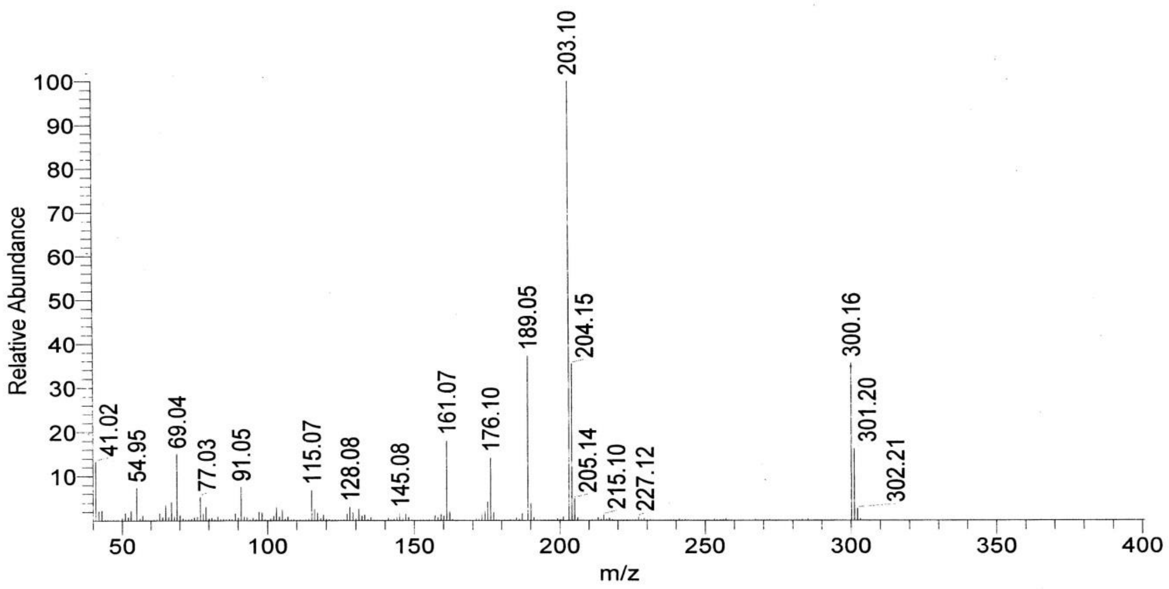

Figure S10. Mass spectrum of compound **4a**

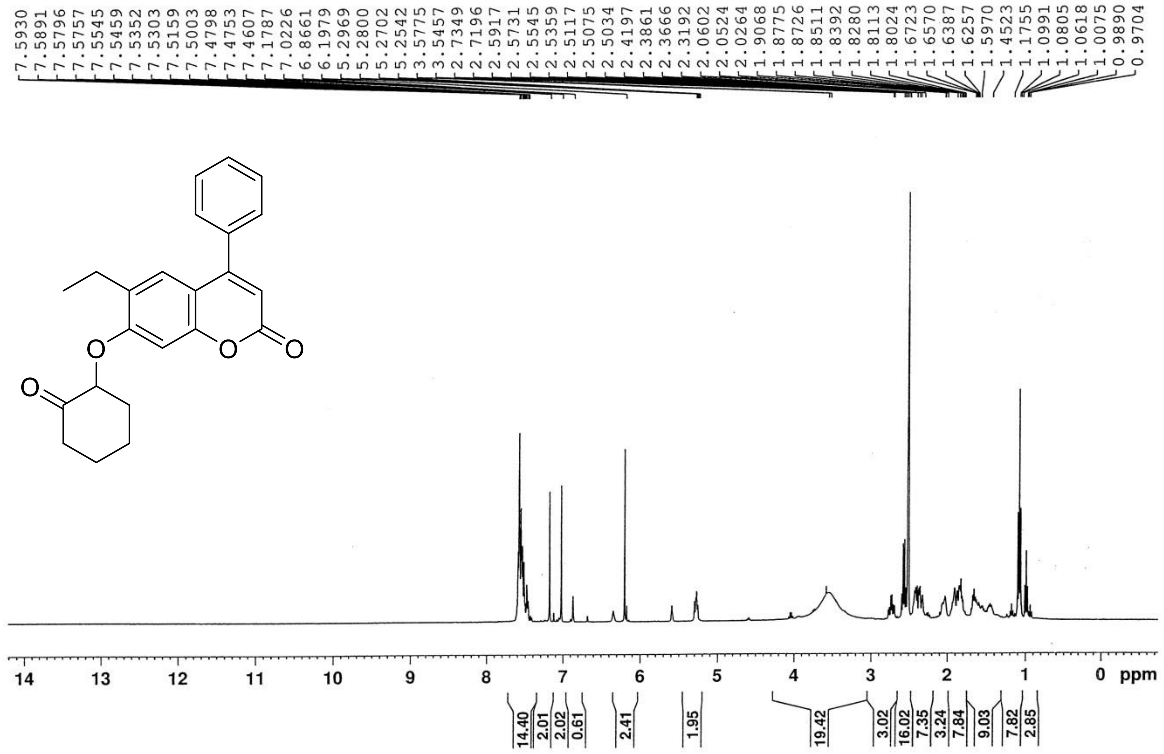

.**Figure S11.** ^{1}H NMR spectrum (400 MHz, DMSO-d_6) of compound **4b**

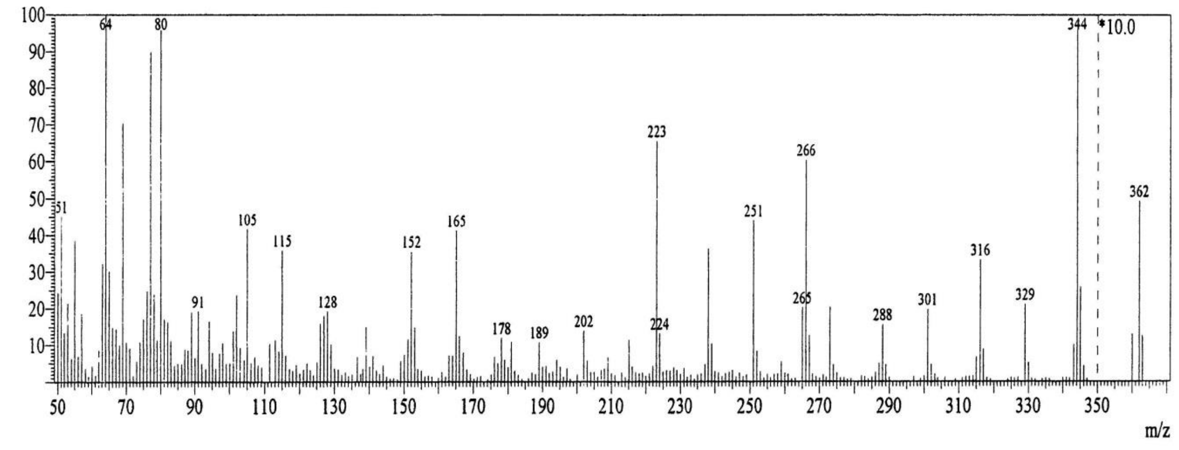

Figure S12. Mass spectrum of compound **4b**

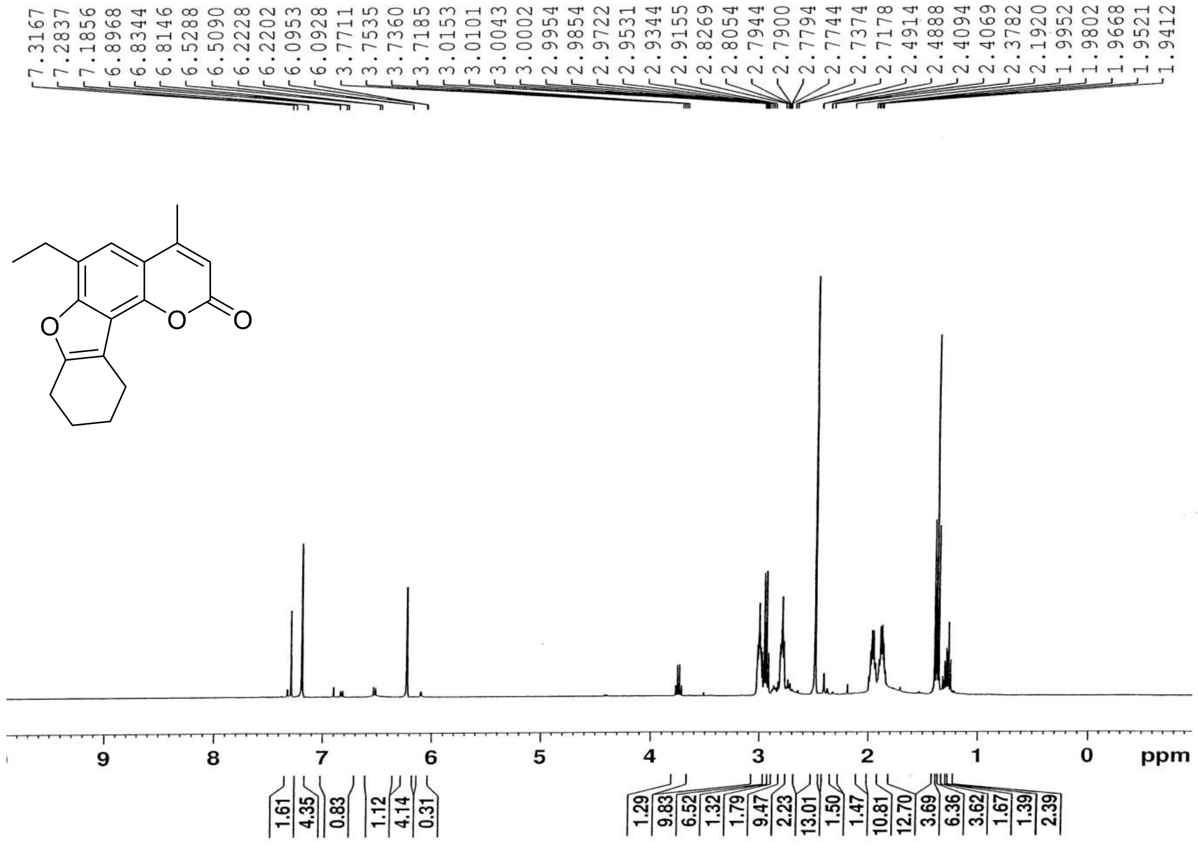

Figure S13. ^{1}H NMR spectrum (400 MHz, $CDCl_3$) of compound **5a**

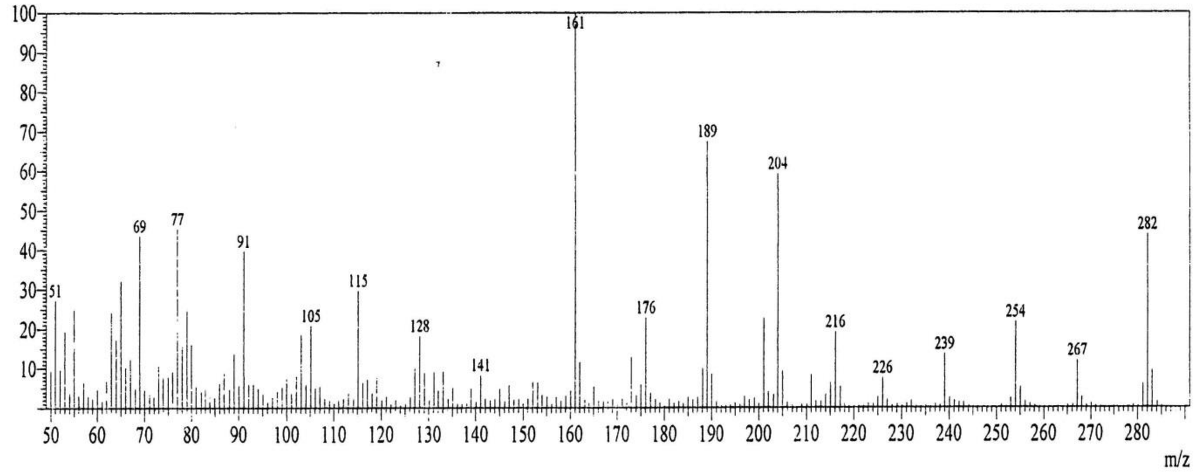

Figure S14. Mass spectrum of compound **5a**

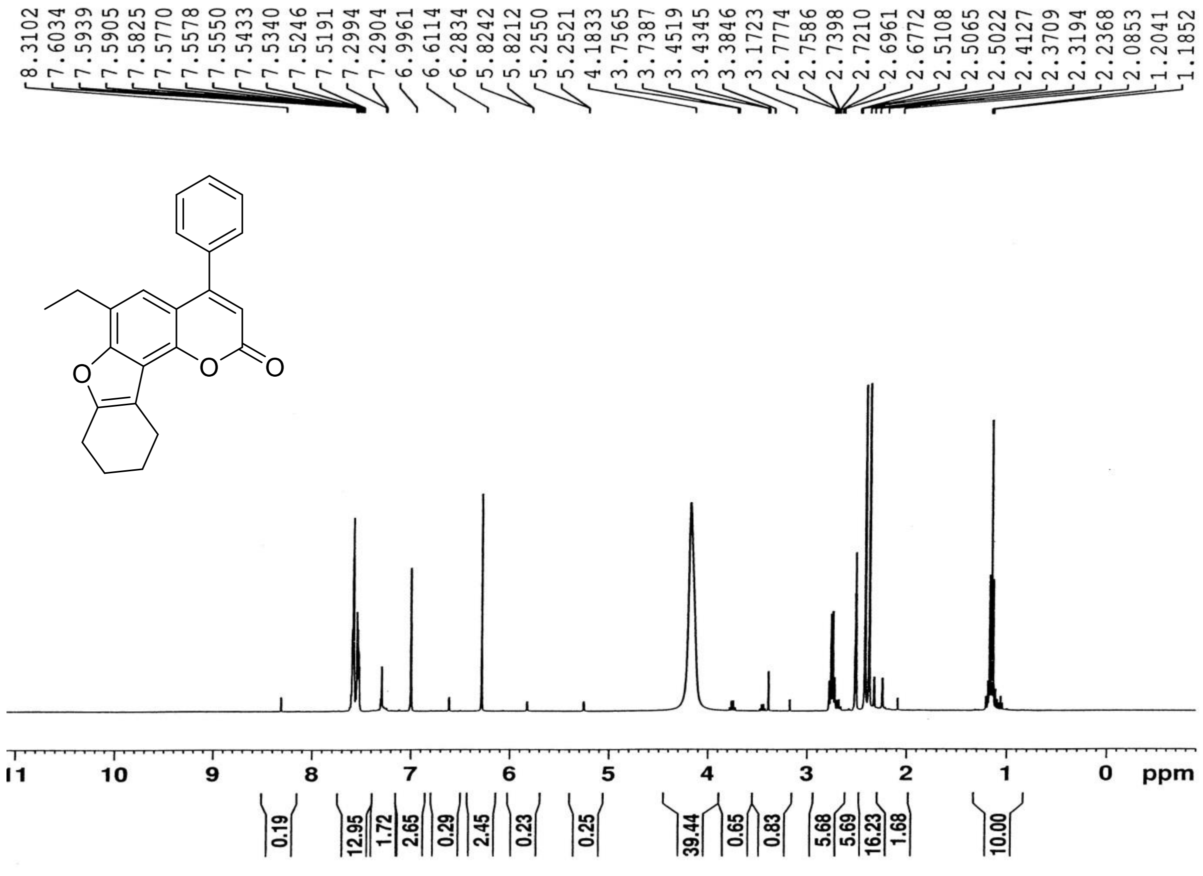

Figure S15. ^{1}H NMR spectrum (400 MHz, DMSO-d_6) of compound **5b**

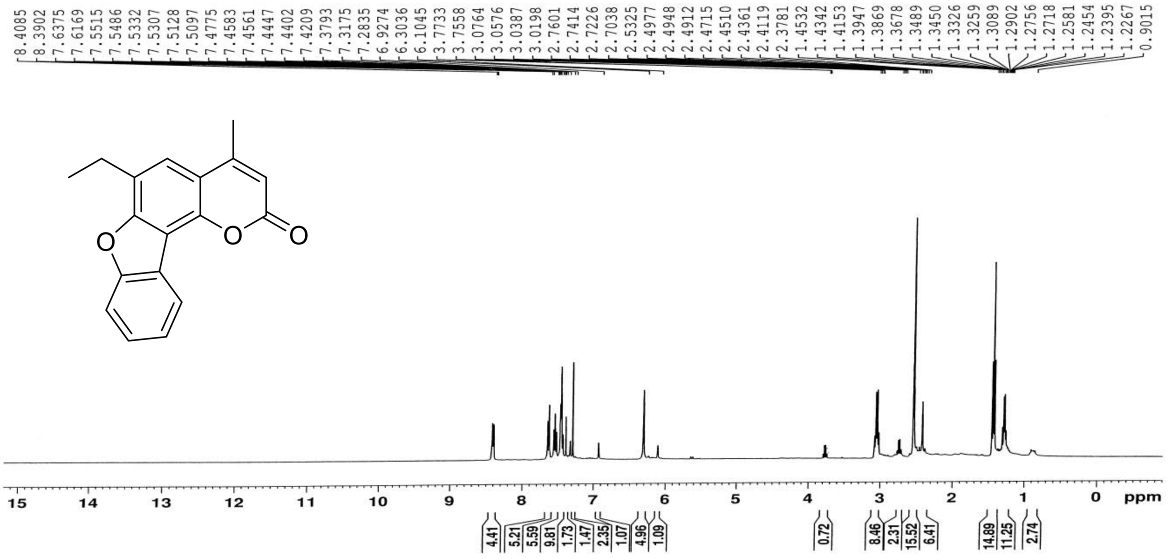

Figure S16. ^{1}H NMR spectrum (400 MHz, $CDCl_3$) of compound **6a**

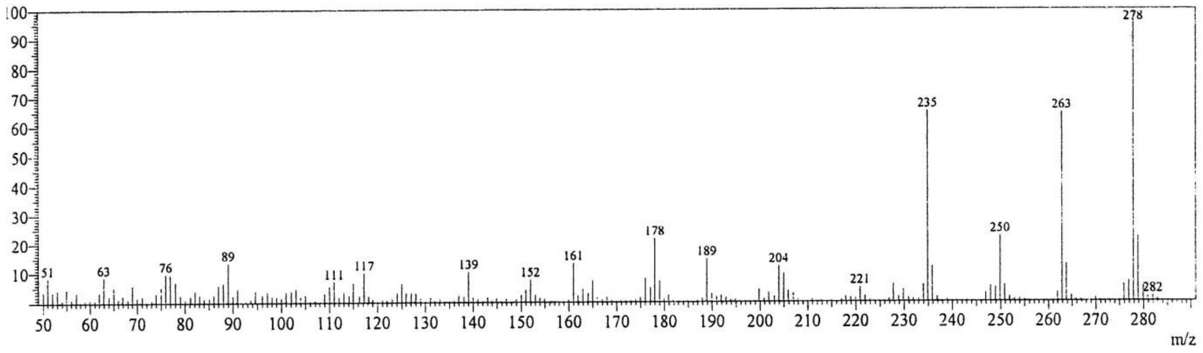

Figure S17. Mass spectrum of compound **6a**

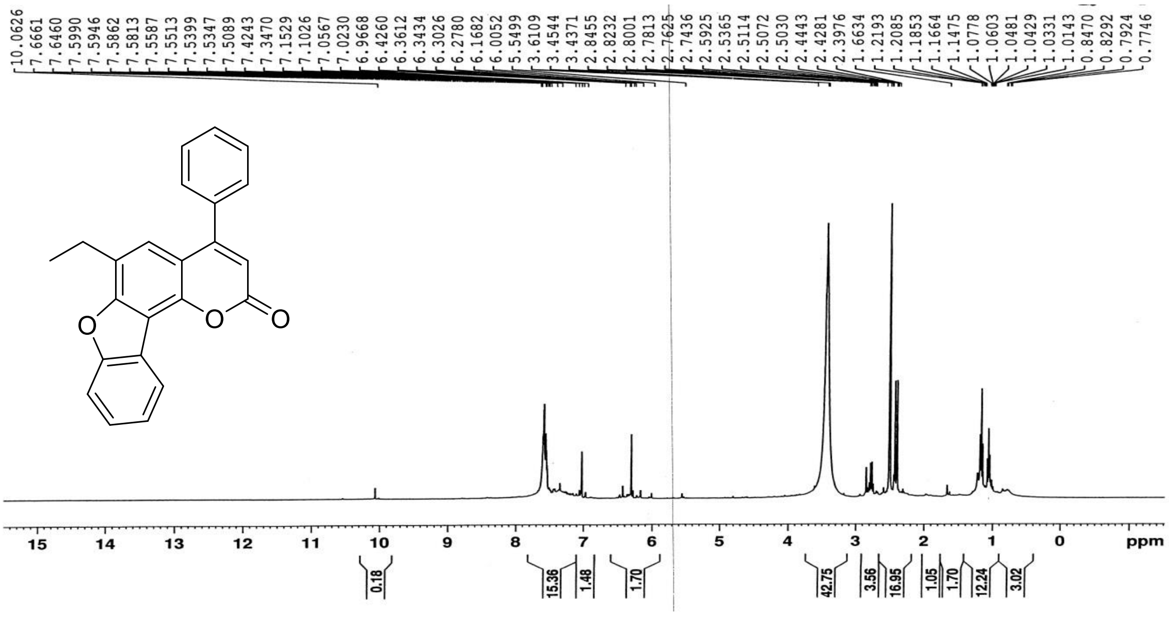

Figure S18. ^{1}H NMR spectrum (400 MHz, DMSO-d_6) of compound **6b**

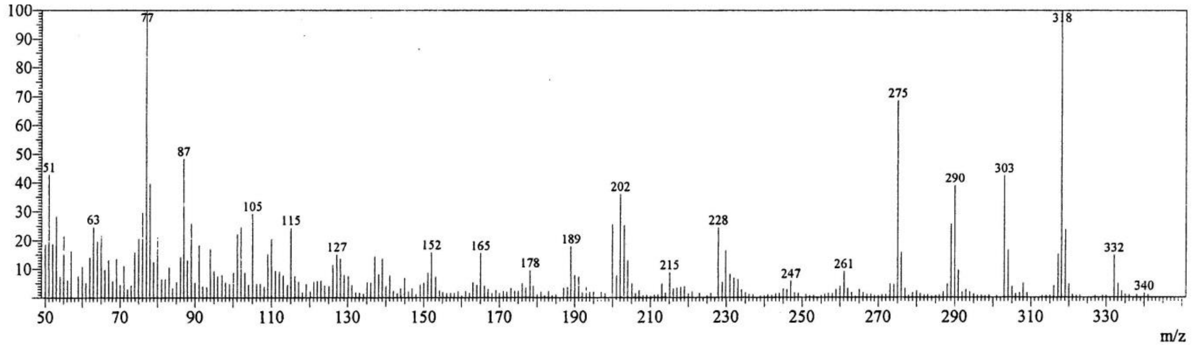

Figure S19. Mass spectrum of compound **6b**

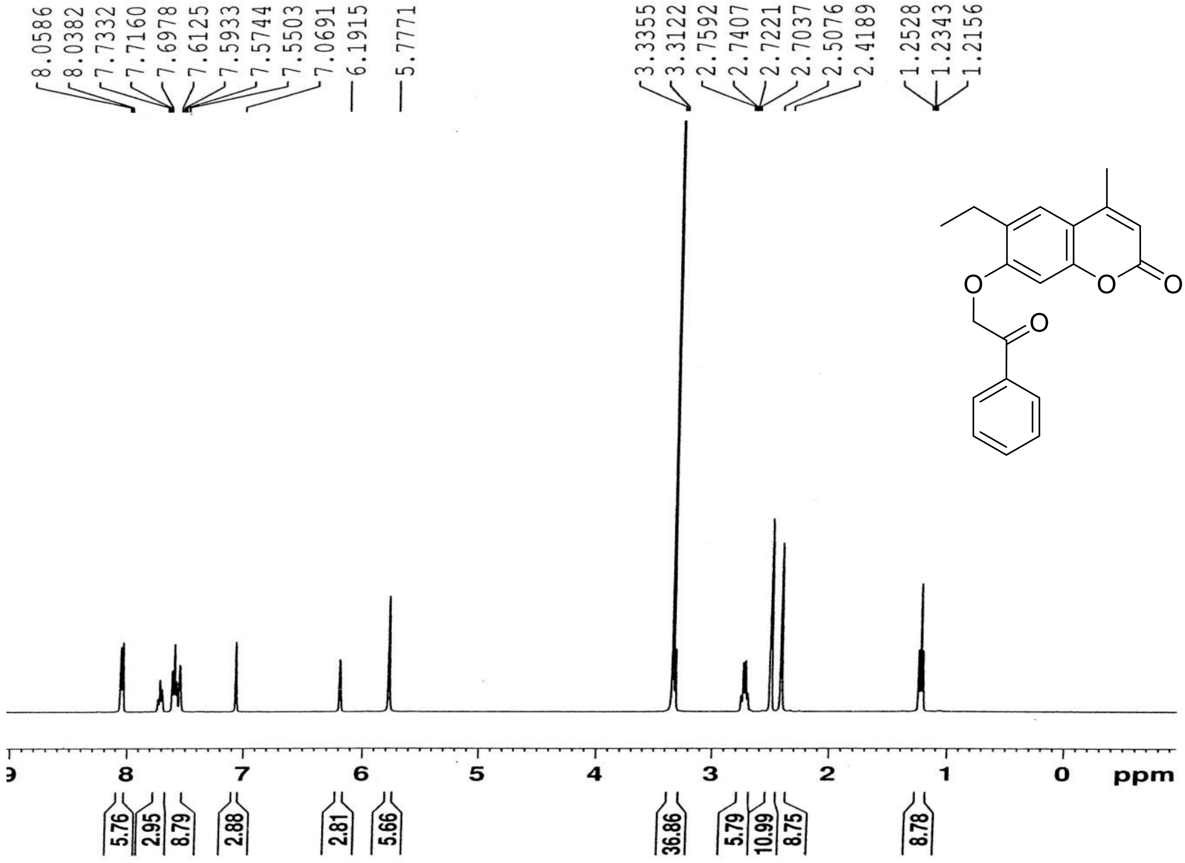

Figure S20. ^{1}H NMR spectrum (400 MHz, DMSO-d_6) of compound **7a**

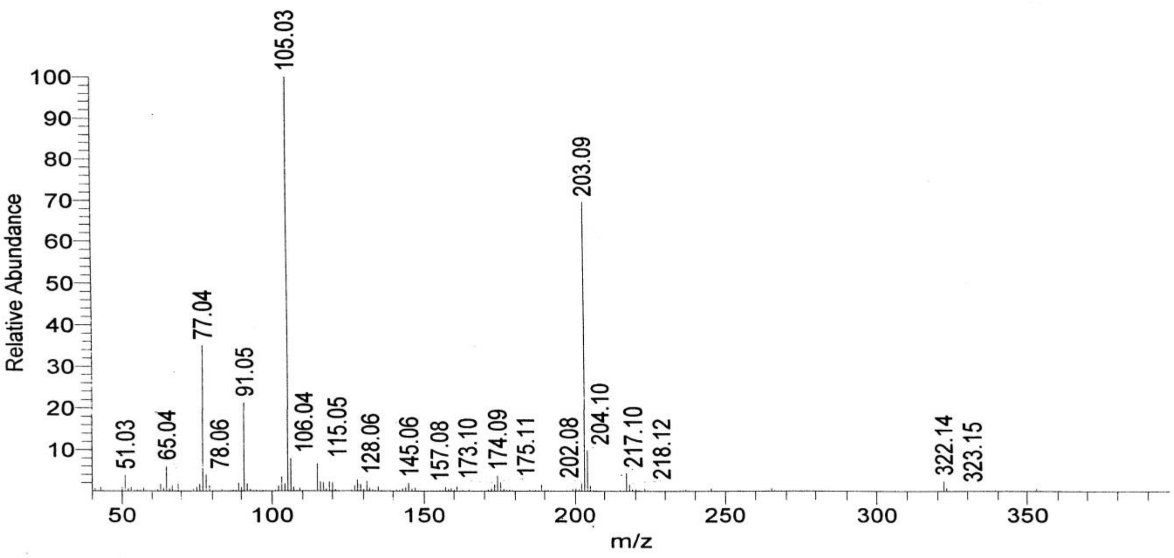

Figure S21. Mass spectrum of compound **7a**

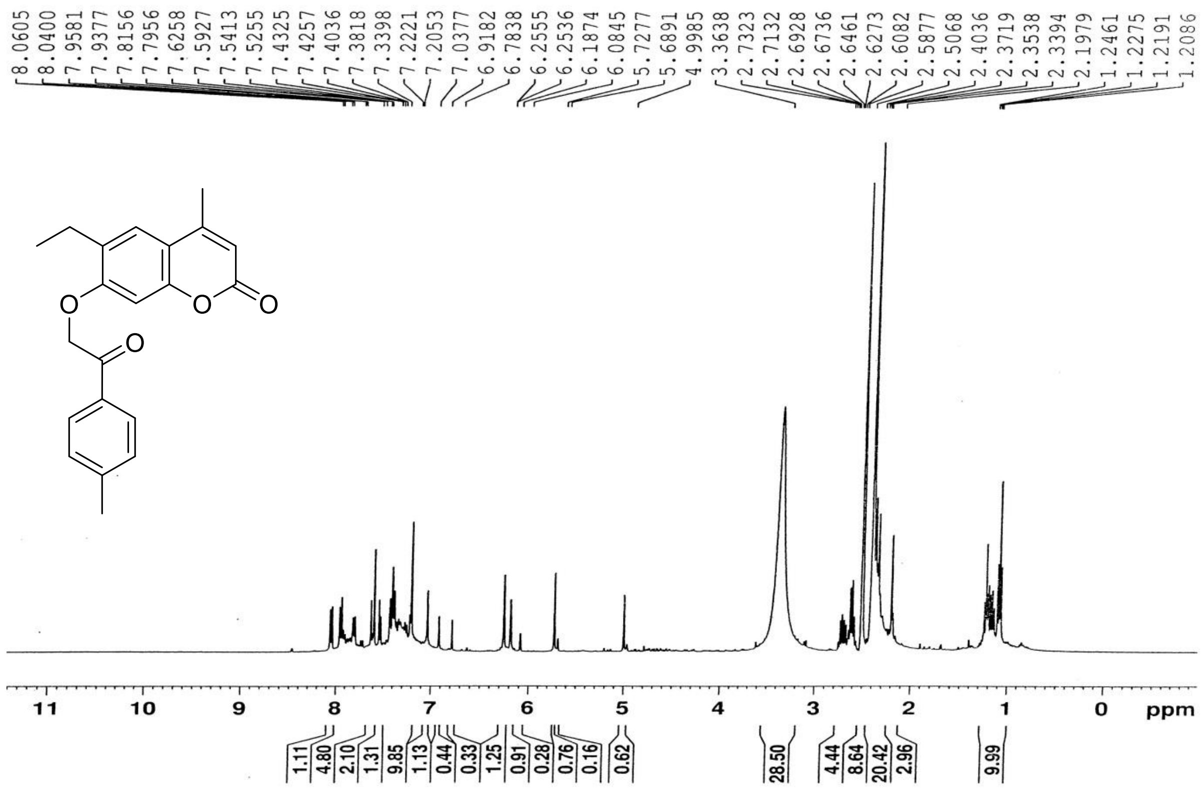

Figure S22. ^{1}H NMR spectrum (400 MHz, DMSO-d_6) of compound **7b**

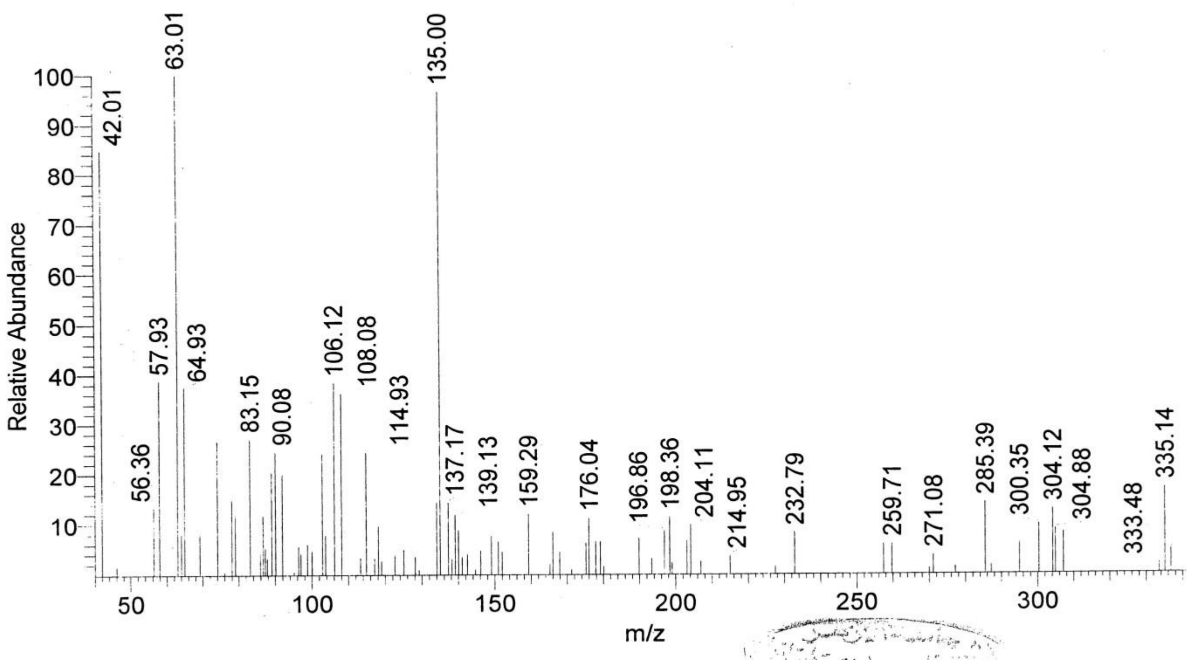

Figure S23. Mass spectrum of compound **7b**

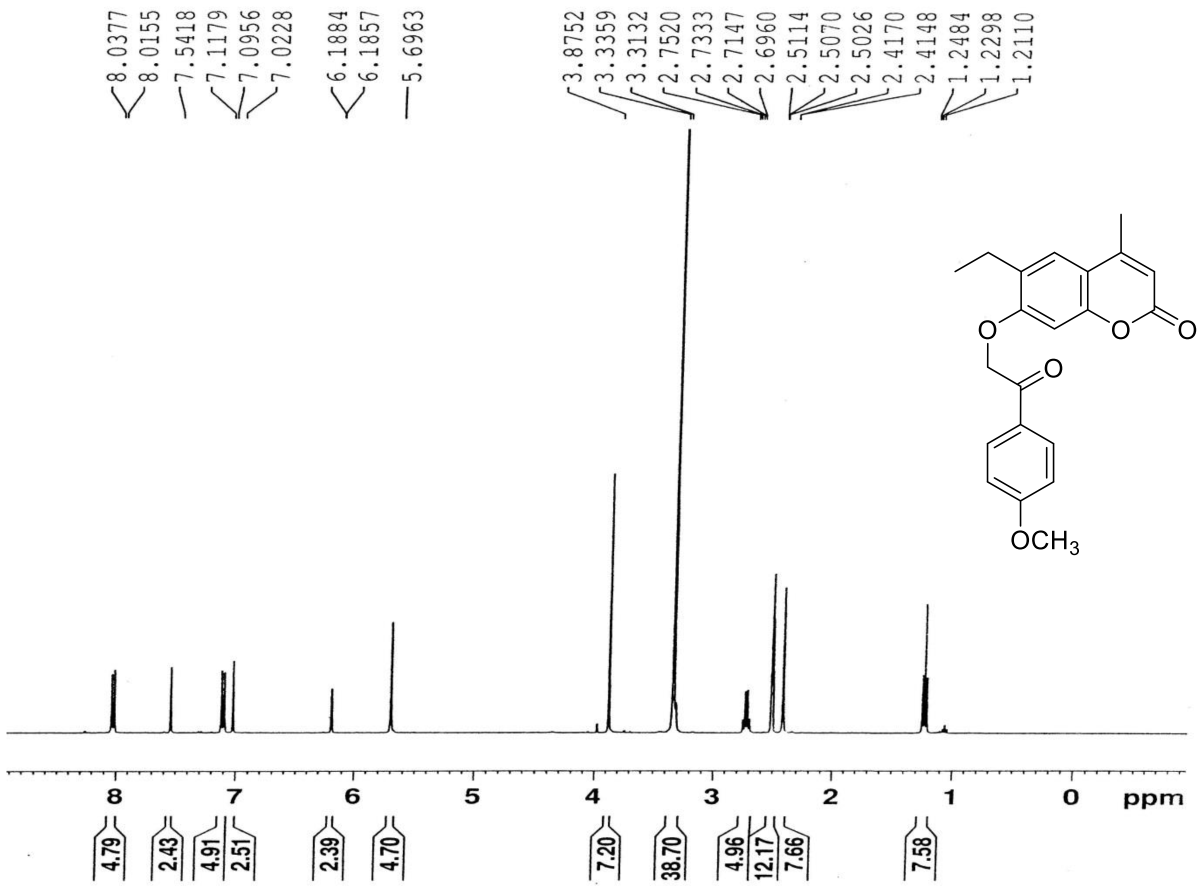

Figure S24. ^{1}H NMR spectrum (400 MHz, DMSO-d_6) of compound **7c**

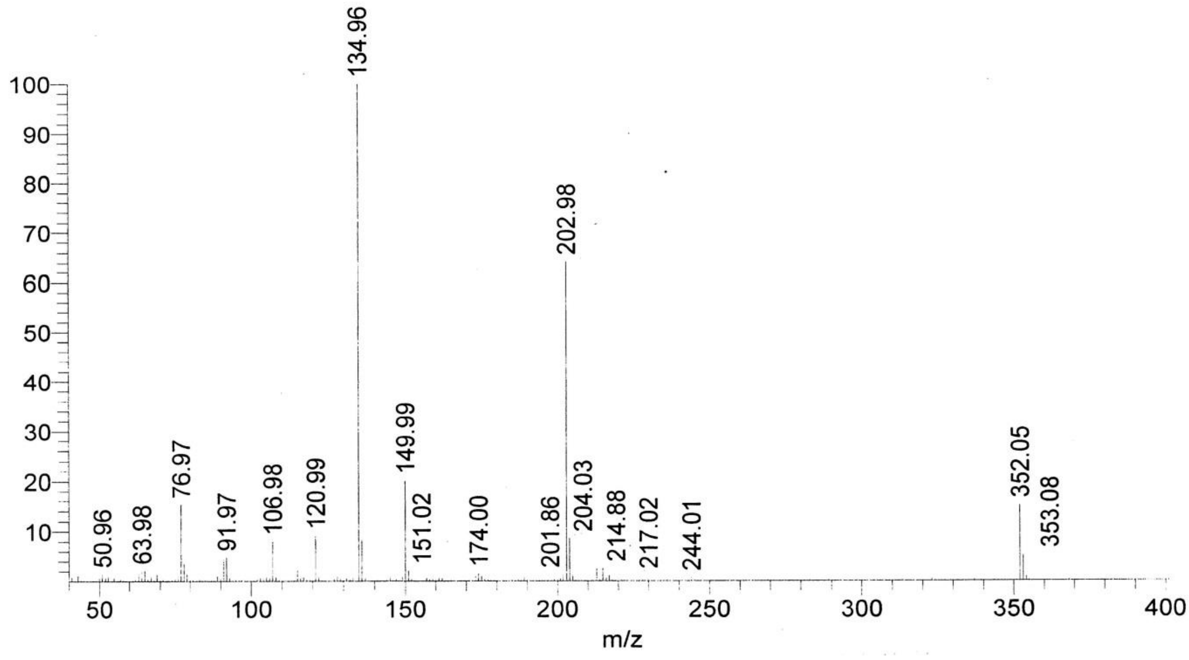

Figure S25. Mass spectrum of compound **7c**

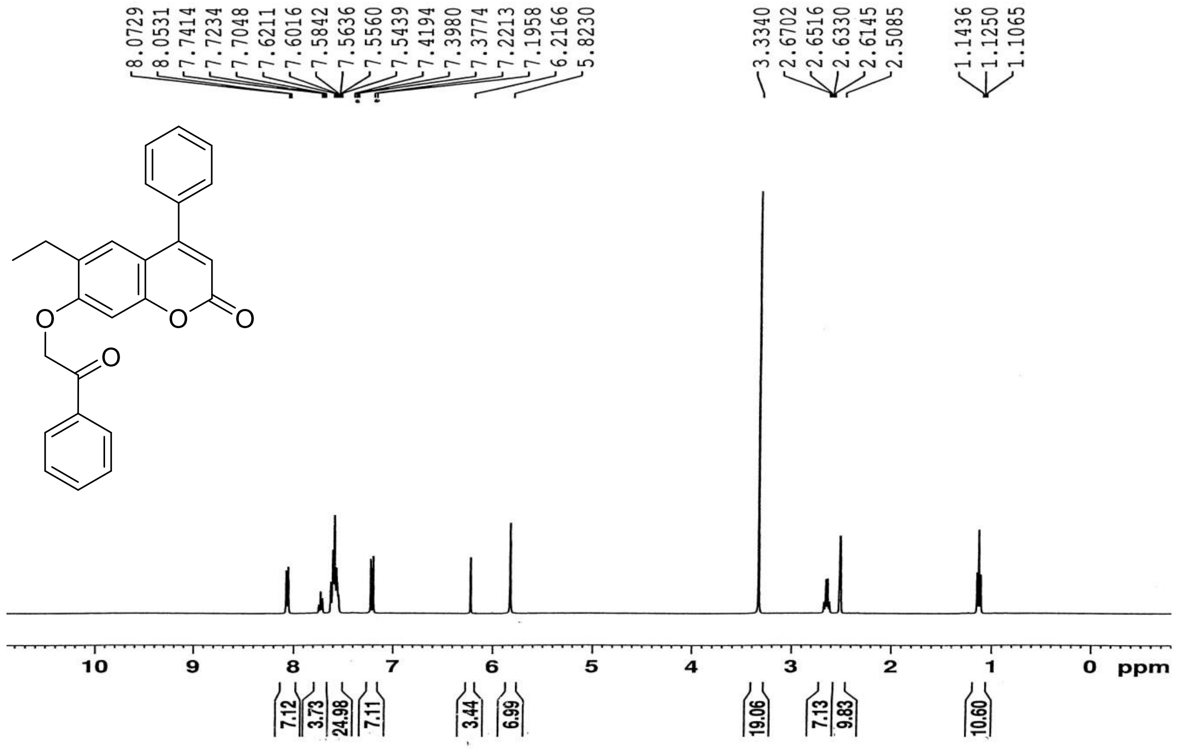

Figure S26. ^{1}H NMR spectrum (400 MHz, DMSO-d_6) of compound **7d**

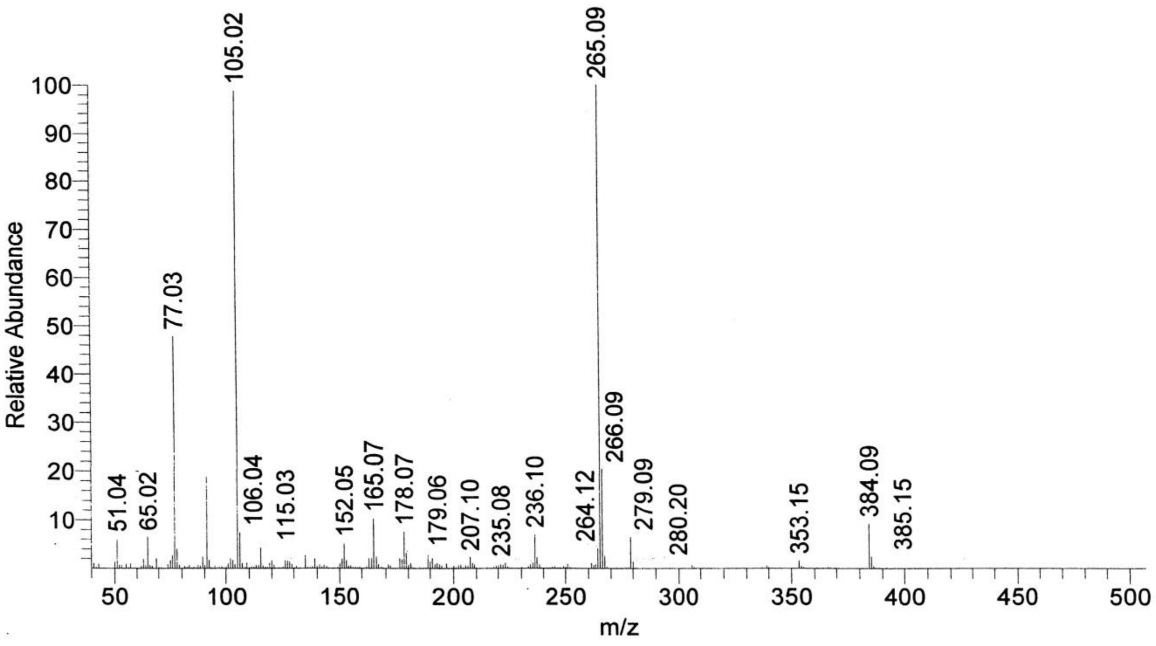

Figure S27. Mass spectrum of compound **7d**

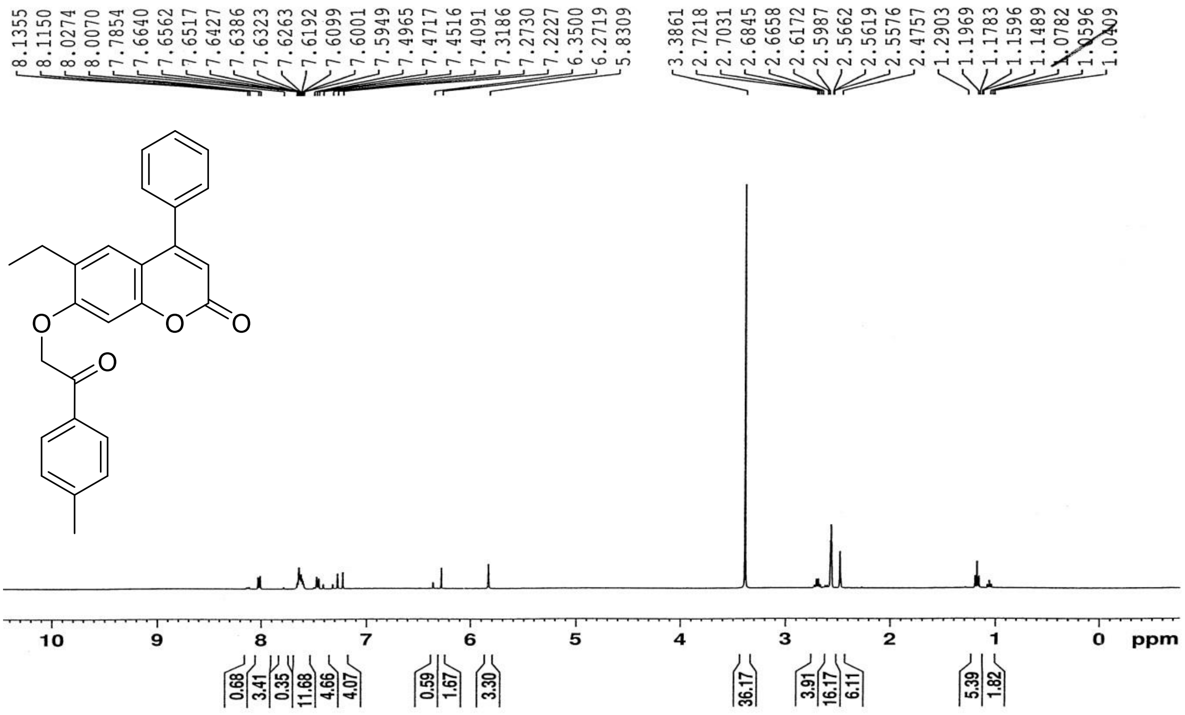

Figure S28. ^{1}H NMR spectrum (400 MHz, DMSO-d_6) of compound **7e**

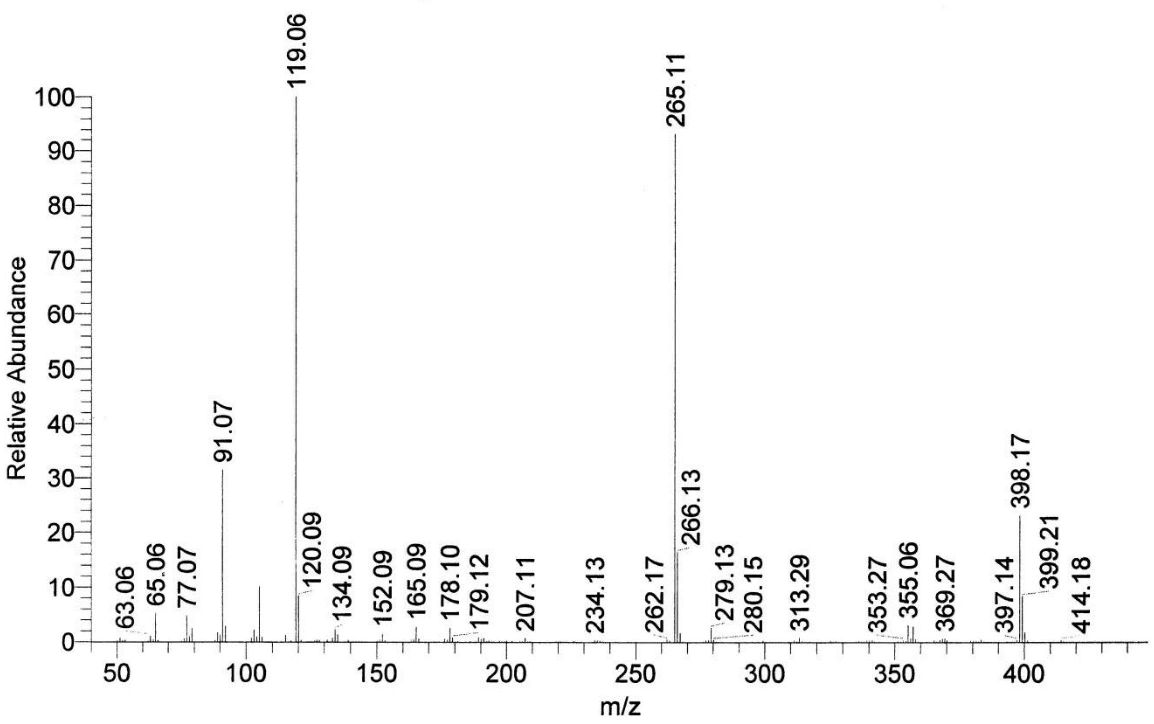

Figure S29. Mass spectrum of compound **7e**

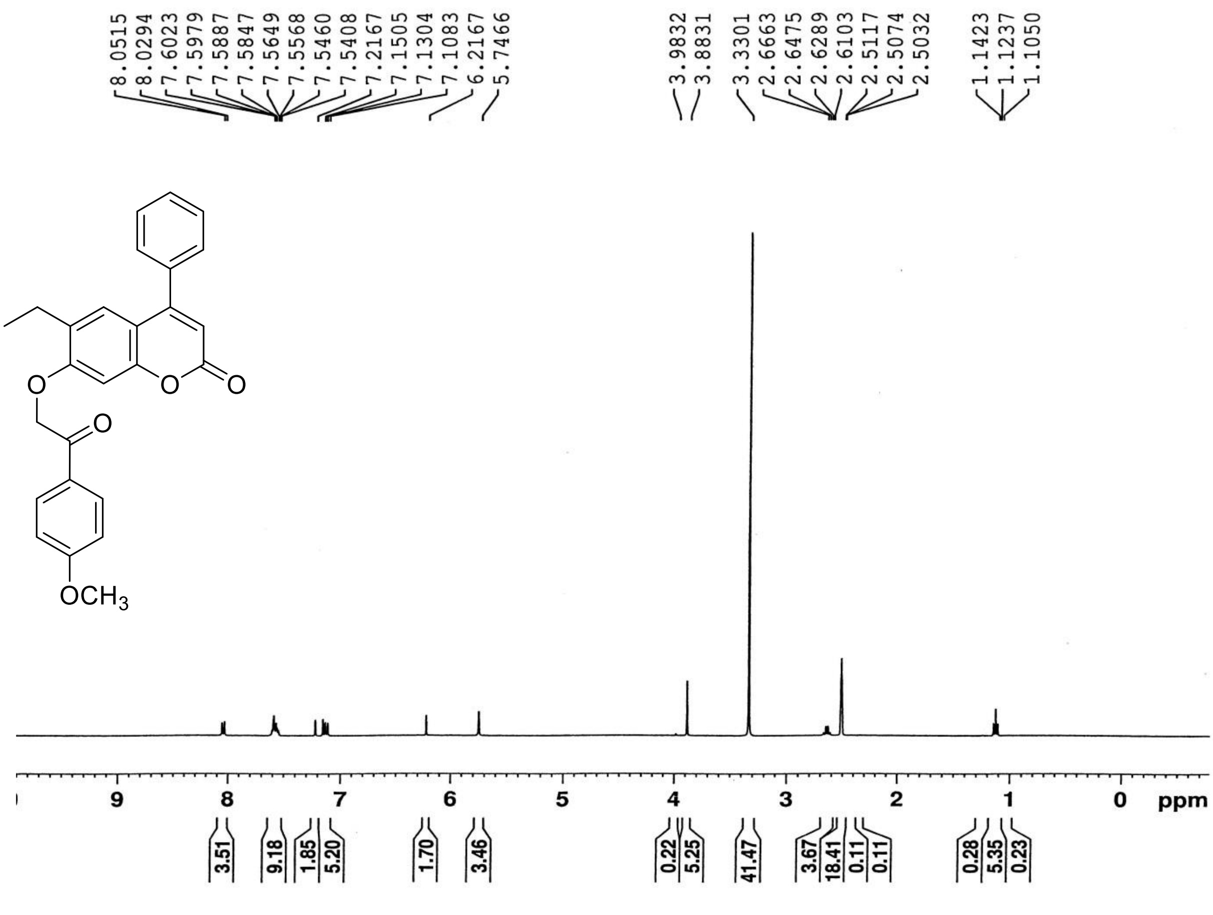

Figure S30. ^{1}H NMR spectrum (400 MHz, DMSO-d_6) of compound **7f**

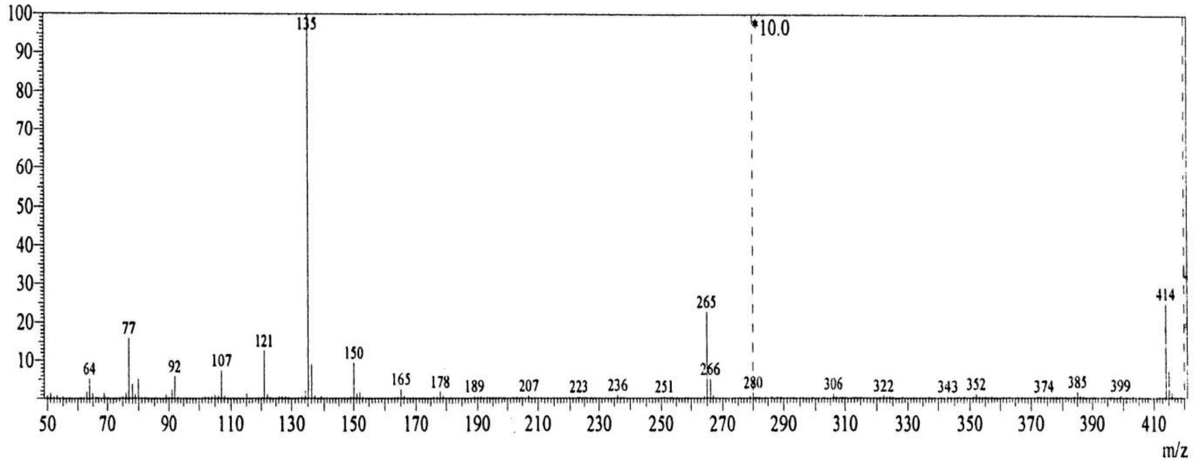

Figure S31. Mass spectrum of compound **7f**

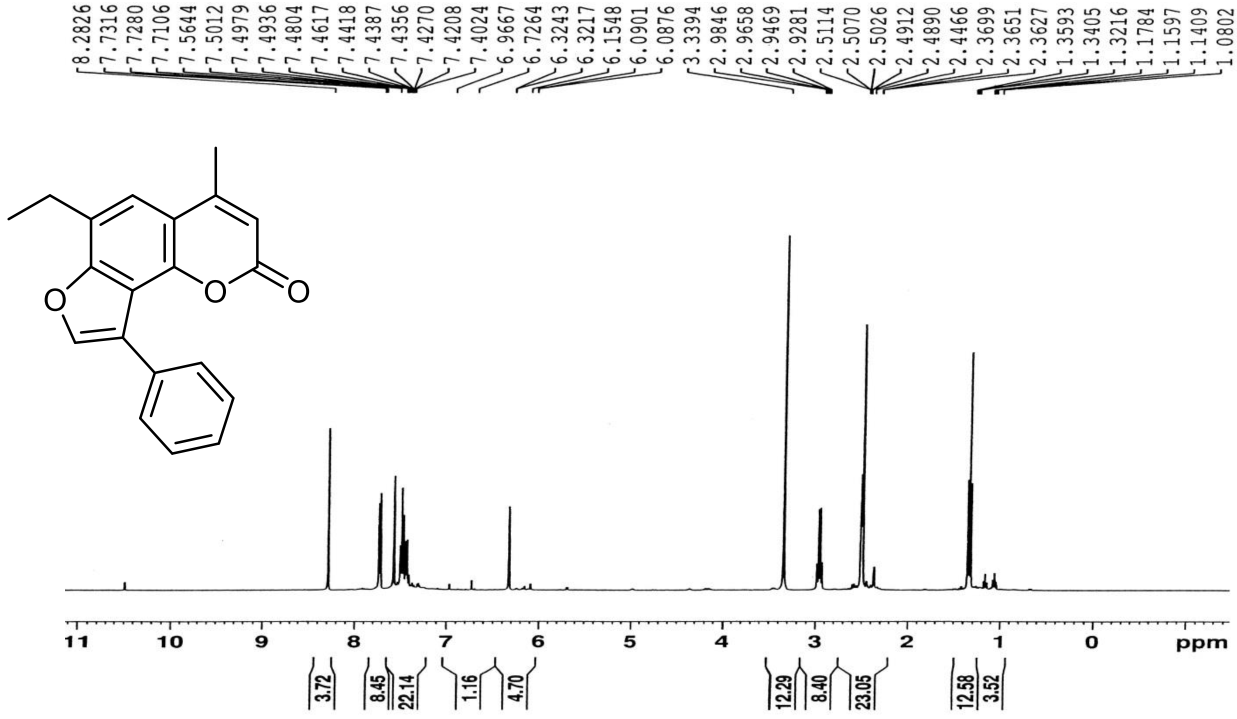

Figure S32. ^{1}H NMR spectrum (400 MHz, DMSO-d_6) of compound **8a**

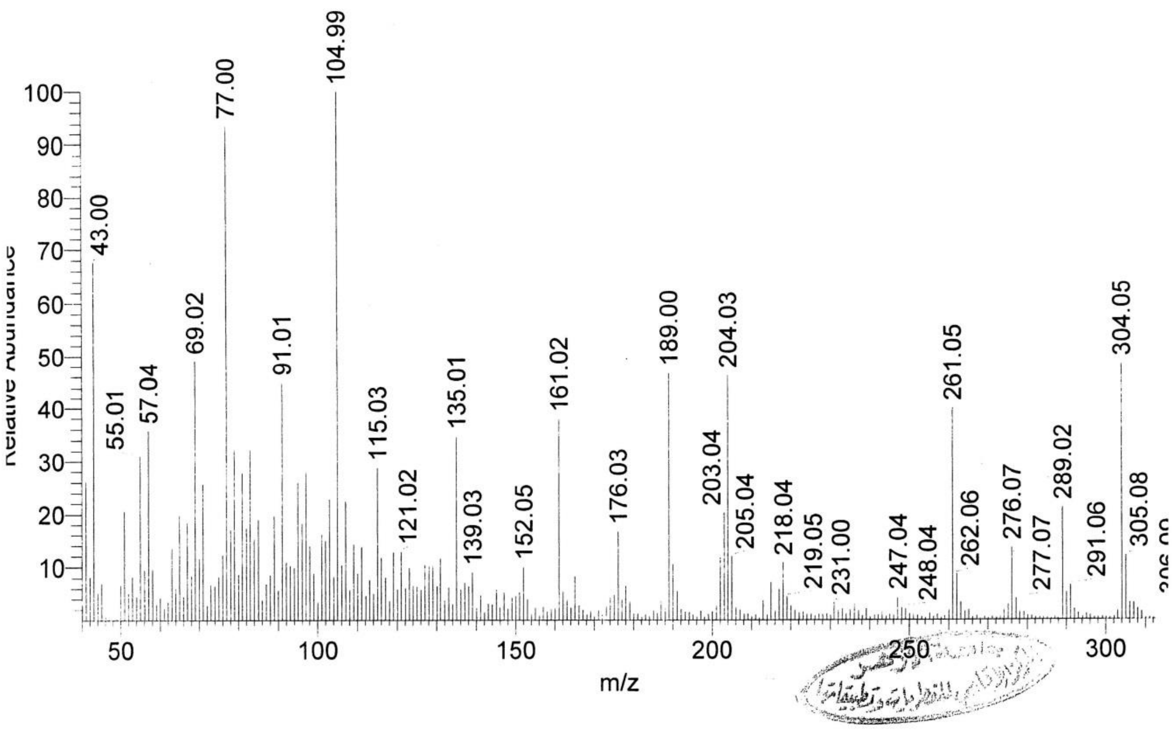

Figure S33. Mass spectrum of compound **8a**

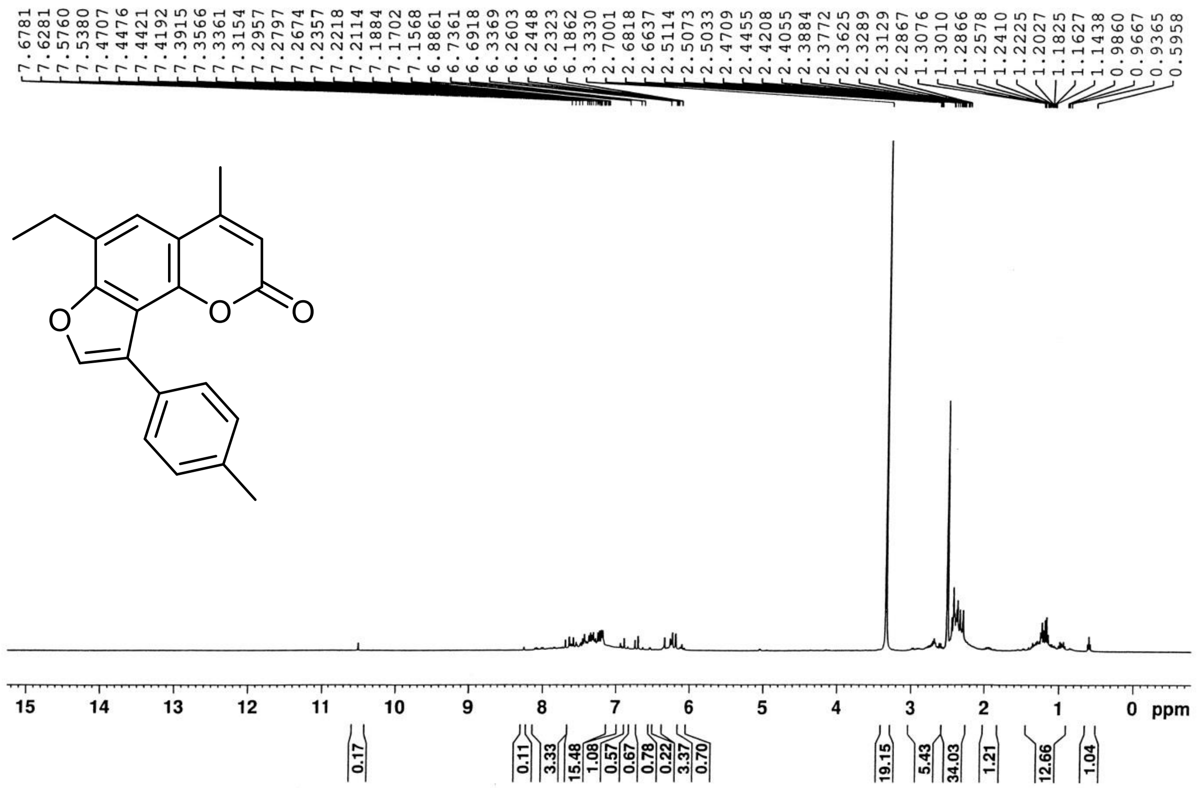

Figure S34. ^{1}H NMR spectrum (400 MHz, DMSO-d_6) of compound **8b**

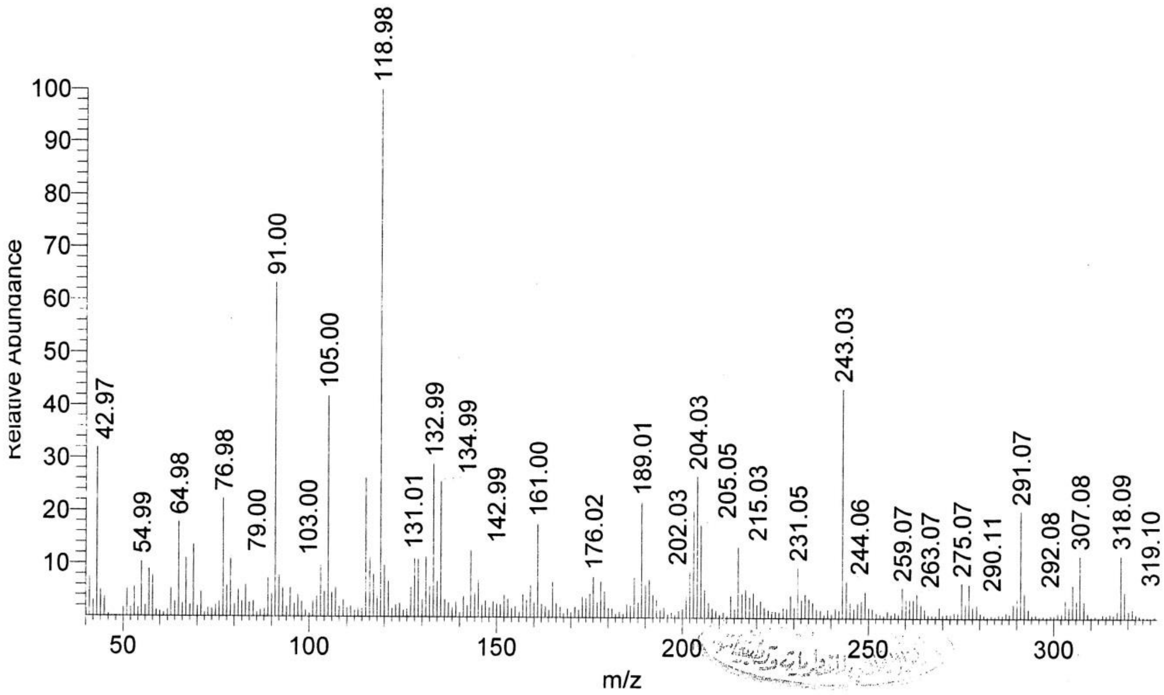

Figure S35. Mass spectrum of compound **8b**

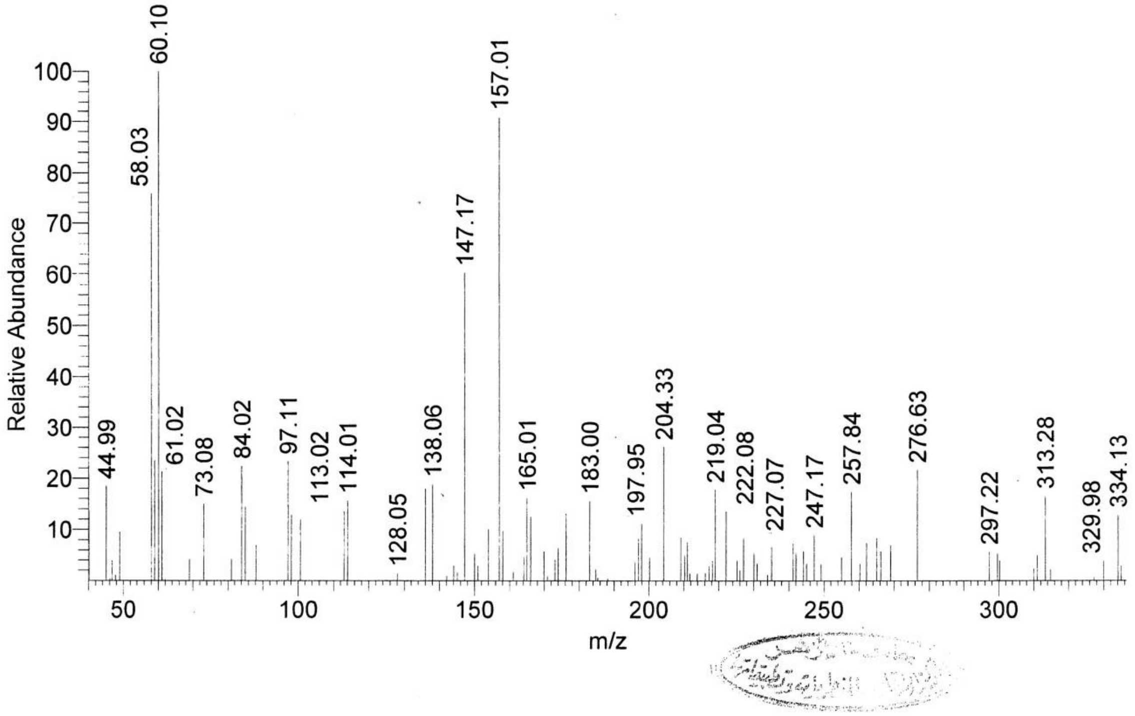

Figure S36. Mass spectrum of compound **8c**

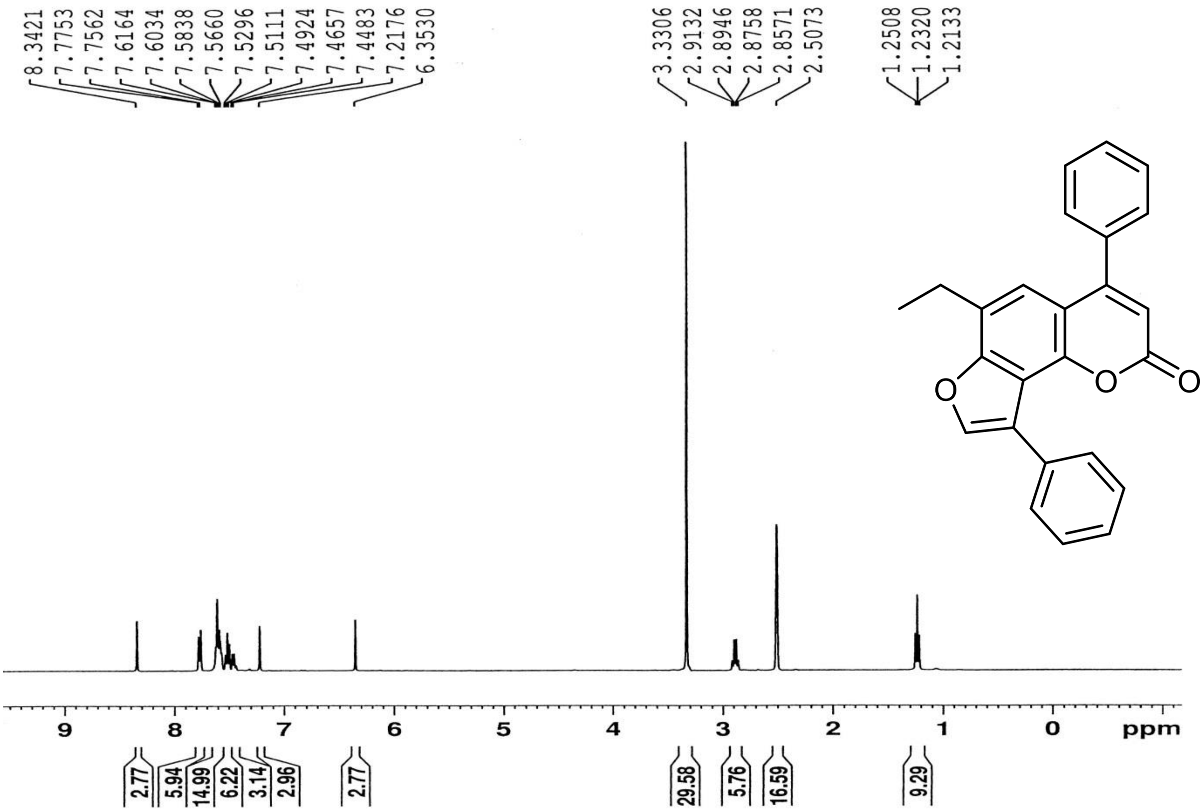

Figure S37. ^{1}H NMR spectrum (400 MHz, DMSO-d_6) of compound **8d**

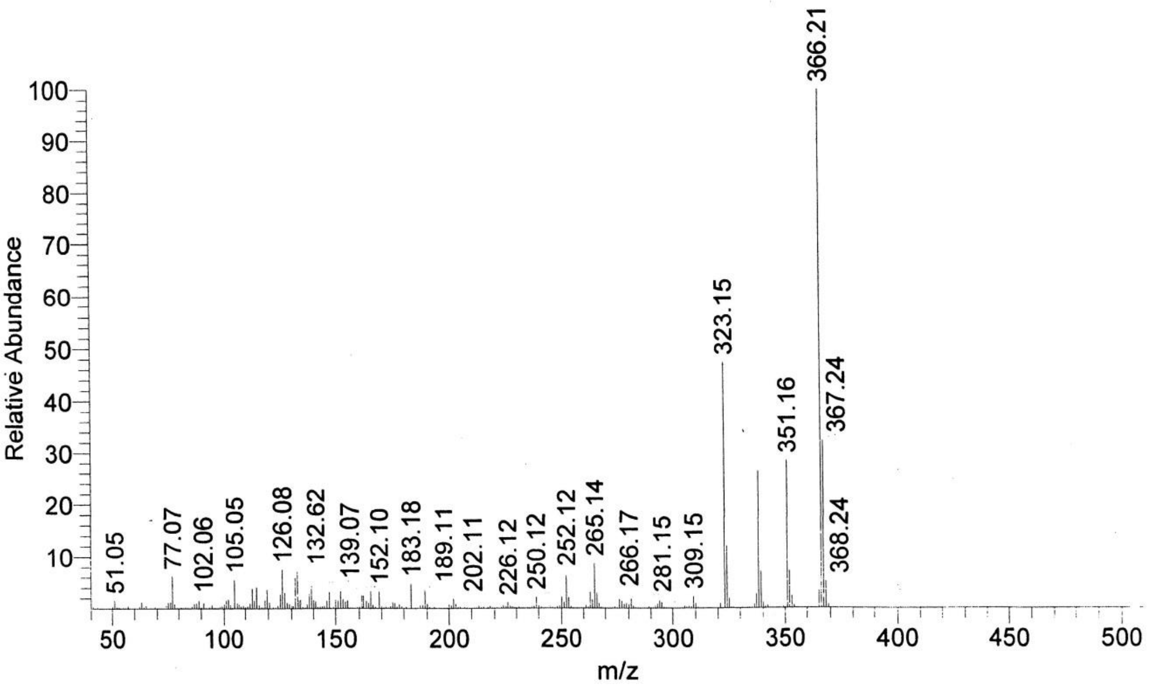

Figure S38. Mass spectrum of compound **8d**

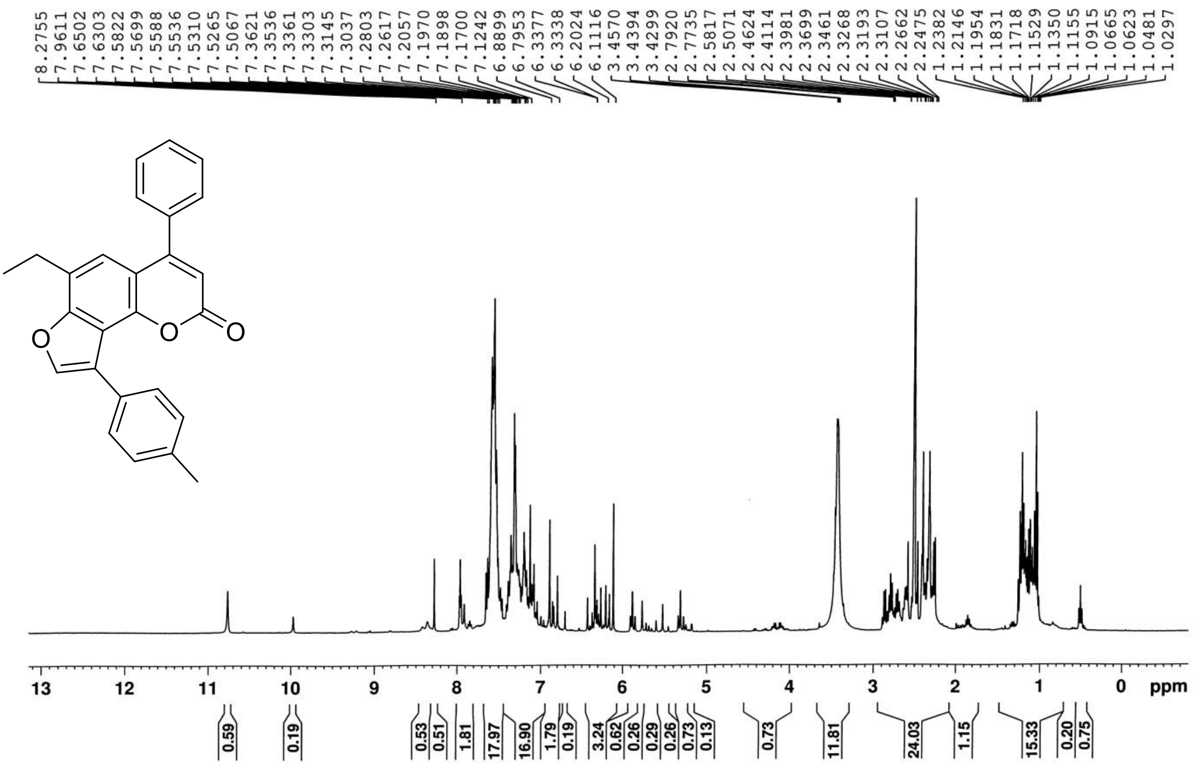

Figure S39. ^{1}H NMR spectrum (400 MHz, DMSO-d_6) of compound **8e**

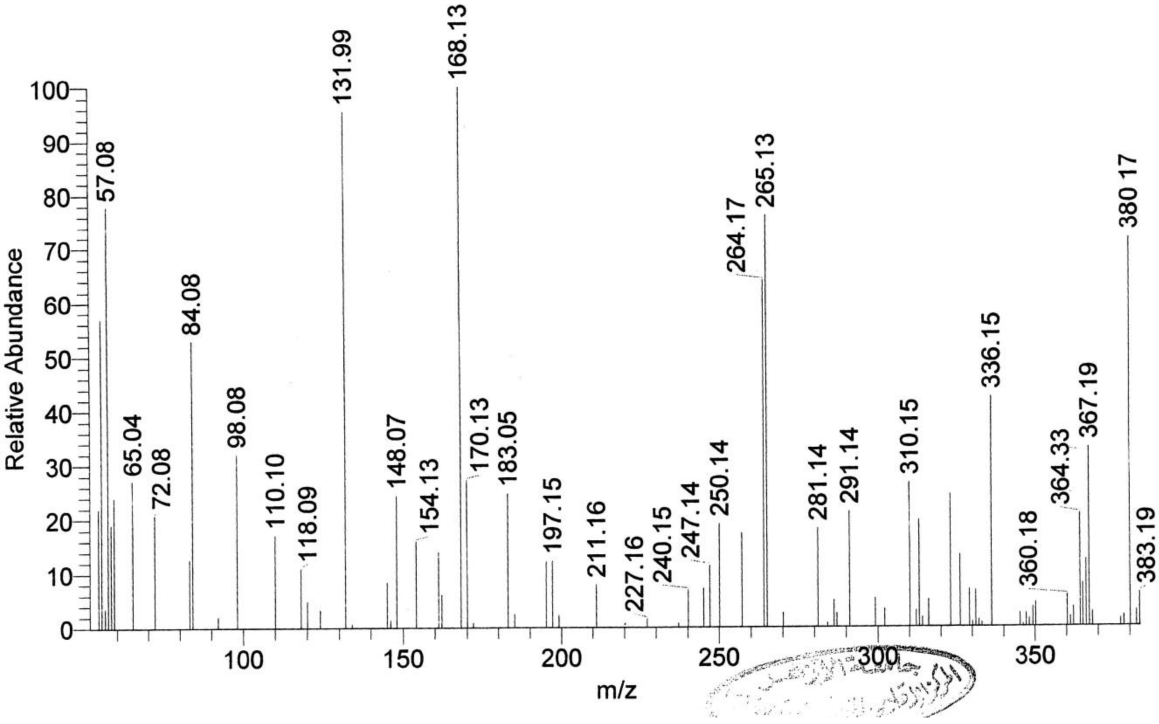

Figure S40. Mass spectrum of compound **8e**

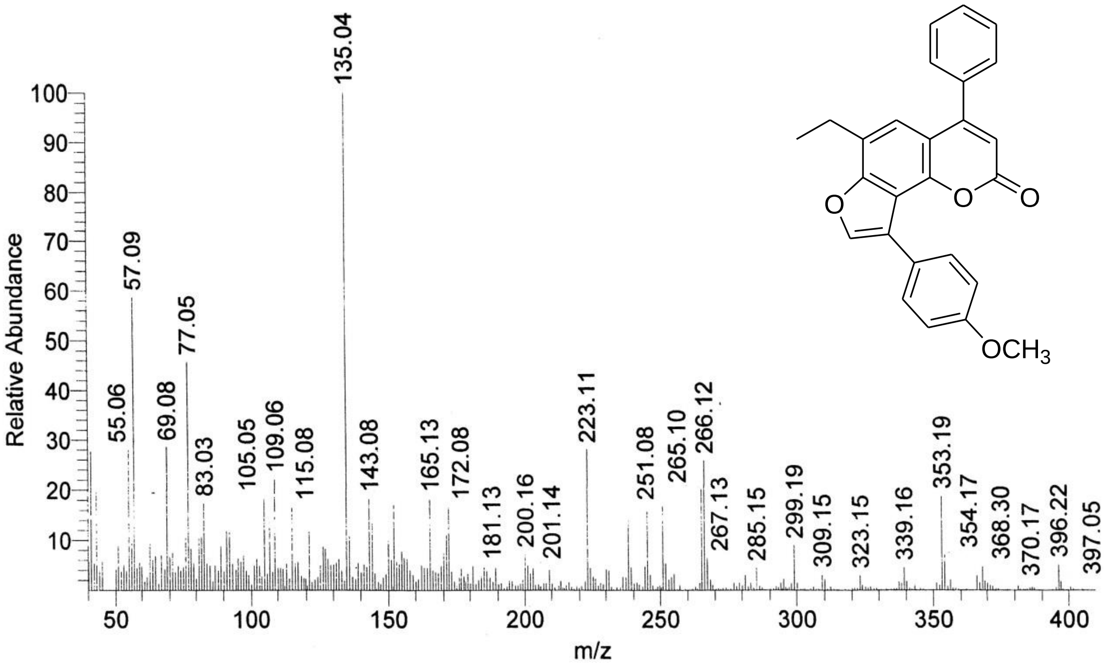

Figure S41. Mass spectrum of compound **8f**
